# Enhanced synaptic transmission at the squid giant synapse by artificial seawater based on physically modified saline

**DOI:** 10.3389/fnsyn.2014.00002

**Published:** 2014-02-12

**Authors:** Soonwook Choi, Eunah Yu, Guilherme Rabello, Suelen Merlo, Ajmal Zemmar, Kerry D. Walton, Herman Moreno, Jorge E. Moreira, Mutsuyuki Sugimori, Rodolfo R. Llinás

**Affiliations:** ^1^Marine Biological LaboratoryWoods Hole, MA, USA; ^2^Department of Neuroscience and Physiology, New York University School of MedicineNew York, NY, USA; ^3^Department of Cell and Molecular Biology, Riberão Preto School of Medicine, University of São PauloRibeirão Preto, Brazil; ^4^Departments of Neurology and Physiology/Pharmacology, The Robert F. Furchgott Center for Neural and Behavioral Science, SUNY Downstate Medical CenterBrooklyn, NY, USA

**Keywords:** neurotransmitter release, squid giant synapse, synaptic transmission optimization, physically modified water, calcium current

## Abstract

Superfusion of the squid giant synapse with artificial seawater (ASW) based on isotonic saline containing oxygen nanobubbles (RNS60 ASW) generates an enhancement of synaptic transmission. This was determined by examining the postsynaptic response to single and repetitive presynaptic spike activation, spontaneous transmitter release, and presynaptic voltage clamp studies. In the presence of RNS60 ASW single presynaptic stimulation elicited larger postsynaptic potentials (PSP) and more robust recovery from high frequency stimulation than in control ASW. Analysis of postsynaptic noise revealed an increase in spontaneous transmitter release with modified noise kinetics in RNS60 ASW. Presynaptic voltage clamp demonstrated an increased EPSP, without an increase in presynaptic ICa^++^ amplitude during RNS60 ASW superfusion. Synaptic release enhancement reached stable maxima within 5–10 min of RNS60 ASW superfusion and was maintained for the entire recording time, up to 1 h. Electronmicroscopic morphometry indicated a decrease in synaptic vesicle density and the number at active zones with an increase in the number of clathrin-coated vesicles (CCV) and large endosome-like vesicles near junctional sites. Block of mitochondrial ATP synthesis by presynaptic injection of oligomycin reduced spontaneous release and prevented the synaptic noise increase seen in RNS60 ASW. After ATP block the number of vesicles at the active zone and CCV was reduced, with an increase in large vesicles. The possibility that RNS60 ASW acts by increasing mitochondrial ATP synthesis was tested by direct determination of ATP levels in both presynaptic and postsynaptic structures. This was implemented using luciferin/luciferase photon emission, which demonstrated a marked increase in ATP synthesis following RNS60 administration. It is concluded that RNS60 positively modulates synaptic transmission by up-regulating ATP synthesis, thus leading to synaptic transmission enhancement.

## Introduction

Determining the biological variables that control both electrical and chemical synaptic transmission between nerve cells, or between nerve terminals and muscular or glandular systems, has been a very significant area of physiological exploration over the decades. Chemical synaptic transmission has had the added attraction of addressing both the transmission gain of the event, as well as the excitatory or inhibitory nature of the junction and its activity-dependent potentiation or depression.

In the present study, we address the effect of artificial seawater (ASW) based on RNS60 (Khasnavis et al., 2012; Mondal et al., 2012), a physically modified isotonic saline that has been altered to include charge-stabilized nanostructures with an oxygen nanobubble core. RNS60 is a physically modified normal saline solution generated by using a rotor/stator device that incorporates controlled turbulence and Taylor-Couette-Poiseuille (TCP) flow under high oxygen pressure. Briefly, sodium chloride (0.9% NaCl), USP pH 5.6 (4.5–7.0, Hospira), is processed at 4°C with a flow rate of 32 mL/s under 1 atm of oxygen backpressure (7.8 mL/s gas flow rate) while maintaining a rotor speed of 3450 rpm. These conditions generate a strong shear layer at the interface between the vapor and liquid phases near the rotor cavities, which correlates with the generation of small bubbles from cavitation. The resulting fluid is immediately placed into glass bottles (KG-33 borosilicate glass, Kimble-Chase) and sealed using gray chlorobutyl rubber stoppers (USP class 6, West Pharmaceuticals) to maintain pressure and minimize leachables. When tested after 24 h, the oxygen content was 55 ± 5 ppm. Chemically, RNS60 contains water, sodium chloride, and oxygen, but no active pharmaceutical ingredients.

Recent studies have demonstrated the properties of RNS60 as an immune modulator (Khasnavis et al., 2012; Mondal et al., 2012). RNS60 has been reported to activate PI3K-Akt pathway, which in turn inhibits NFkB activity and production of proinflammatory molecules such as iNOS and IL1β in glial cells (JBC2012). Moreover, RNS60 boosts Tregs through suppression of NO production thus inhibiting the differentiation of Th17 and Th1 cells (Mondal et al., 2012), an important process in the pathophysiology of allergic encephalomyelitis.

We found that ASW based on RNS60 enhances synaptic transmission without producing abnormal excess release as is seen, for example, with CaM kinase II that abnormally increases I_*Ca*_ (Llinas et al., 1991). We then proceeded to address possible mechanisms responsible for such enhancement. RNS60 improves the effectiveness of the synapse within physiological bounds. The degree of improvement varied across synaptic preparations according to the effectiveness of the synaptic transmission at the start of the experiment.

## Materials and methods

### Squid giant synapse preparation

All experiments were carried out at the Marine Biological Laboratory in Woods Hole, MA (MBL). As in previous research with this junction (Katz and Miledi, 1967, 1971; Llinas et al., 1976, 1981a; Augustine and Charlton, 1986), squid (*Loligo paelli*) stellate ganglia were rapidly removed from the mantle under running sea water following decapitation. The isolated ganglion was placed in a recording chamber such that both the presynaptic and postsynaptic terminals could be directly visualized for microelectrode penetration and continuously superfused with ASW. A total of 75 synapses were studied with the number of dissected preparations being close to 150. In fact, many synapses dissected were not usable, as clear anatomical and optimal transparency is required for experimental implementation stability.

### Superfusion solutions

Two standard and one physically modified ASW solutions were used in these experiments. Salts were added to 1 liter of distilled water or a 40 ml bottle of physically modified water such that the final salt composition and pH were identical in every case (423 mM NaCl, KCl 8.27 mM, CaCl_2_ 10 mM, MgCl_2_ 50 mM, buffered to 7.2 with HEPES, salinity 3.121%). ASW made with distilled water or physically modified saline was prepared each day and keep at 4° until the start of the experiment. At the start of an experiment, the control ASW and one 40 ml bottle of RNS60 ASW was removed from the refrigerator, brought to room temperature, and the oxygen content measured. Several synapses (5–15) were dissected and studied each day. All experiments were carried out at room temperature (15–18°C) as is our standard practice.

The physically modified saline was RNS60 ASW, made using RNS60 that contains oxygenated nanobubbles prepared with TCP flow. The standard ASWs were: (1) Control ASW, made using distilled H_2_O with air diffusion oxygenation (without bubbling); and (2) NS30612 ASW made using unprocessed normal saline from the same source solution as used to make RNS60. RNS60 and NS30612 were a gift from Revalesio.

In our initial experiments synaptic transmission in NS30612 was found to be indistinguishable from that recorded in our standard control ASW (not shown); ASW was used as the initial step in all experiments.

### Oxygen content measurements

Oxygen measurement of each superperfusate was determined using a Unisense MicroOptode near infrared (NIR, 760–790 nm) sensing probe (400 μm) corrected for temperature and salinity. The mean and s.e.m. of the oxygen content of each of the ASWs measured over 10 min were: (1) Control ASW 268 ± 0.26 μmol/l (8.57 ppm) (2) RNS60 ASW 878 ± 0.8μmol/l (28.1 ppm); (3) Normal Saline (NS) 266 ± 0.18 μmol/l (8.5 ppm). The oxygen content of RNS60 ASW is quite stable. Over the period of a typical experiment, about 30 min, oxygen content of the RNS60 ASW decreased by about 8.7%.

### Electrophysiological methods

Following stable presynaptic and postsynaptic microelectrode impalement and the demonstration of synaptic transmission following presynaptic electrical stimulation the experimental procedure was initiated. The postsynaptic electrodes were beveled to reduce their resistance (<1 mΩ) and thus improved the signal/noise ratio. To evaluate changes in the RC properties of the postsynaptic membrane, the decay constant of the falling phase of the EPSPs was estimated using a built in curve fit function for a decaying exponential (exp Xoffset, Igor Pro, Wavemetrics, Inc.).

### Evoked synaptic transmission

Glass microelectrodes were inserted into the largest (most distal) presynaptic terminal and the corresponding postsynaptic axon. Evoked presynaptic and postsynaptic action potentials were recorded following our standard protocol (Llinas et al., 1981b). The synapse was activated either by extracellular electrical stimulation of the presynaptic axon via an insulated silver wire electrode pair or by directly depolarizing the presynaptic terminal through a second intracellular current injecting electrode. Nerve stimulation was delivered as single stimulus or a train (250 ms at 200 Hz delivered at 1 Hz).

### Spontaneous synaptic transmission

Spontaneous transmitter release was recorded postsynaptically as noise fluctuation of the postsynaptic membrane potential at the synaptic junction (Lin et al., 1990). Synaptic noise measurements provided a second method to assess synaptic viability, and a probe to understand possible affects of RNS60 on spontaneous synaptic vesicular release kinetics. By combining electrophysiological and ultrastructural analysis, we further assessed vesicular recycling properties on the synapse. This combination together with the use of mitochondrial inhibitors, such as oligomycin, allowed us to study the mechanism of RNS60 action on ATP synthesis (Lardy et al., 1958).

Synaptic noise was recorded using a Neurodata Instrument amplifier (ER-91) with a Butterworth filter (0.1–1 kHz). Noise analysis was based on postsynaptic spontaneous unitary waveform determination via two exponential functions (Verveen and Defelice, 1974), *F*(*t*) = *a*[/[*e*^−*t*/τ *d*^ − *e*^−*t*/τ *r*^] where *a* is an amplitude scaling factor and τ*d* and τ*r* are the decay and rise time constants respectively.

The power spectrum derived from the unitary potentials is *S*(*f*) = 2*na*^2^(τ_*d*_− τ_*r*_)^2^/[1 + 4π^2^*f*^2^τ^2^_*d*_)(1 + 4π^2^*f*^2^τ^2^_*r*_)] where *n* is the rate of unitary release *f* and *a*, τ*d* and τ*r* are the same as above. The change in spontaneous release was quantified by averaging noise amplitude in noise frequencies between 20 and 200 Hz.

In order to address the noise fluctuation changes observed following RNS60 based ASW we implemented a numerical solution for the noise profile (Lin et al., 1990). As in previous studies (Lin et al., 1990), the time constant for the miniature potential rise time was determined as having a 0.2 ms and the fall time as 1.5 ms. The noise results following RNS60 were found to have a rise time of 0.2 and a fall time of 2.5 ms. The parameters for the RNS60 noise profile were selected by goodness of fit.

### Presynaptic voltage clamp

The voltage clamp experiments followed our standard protocol (Llinas et al., 1981a). Briefly, two glass micropipette electrodes were inserted into the most distal presynaptic terminal digit at the synaptic junction site and a third micropipette impaled the postsynaptic axon at the junction site. One of the presynaptic electrodes was used for current feedback delivery supporting the voltage clamp loop, while the second monitored membrane potential. Presynaptic voltage was measured using an FET input operational amplifier (Analog Devices model 515, Analog Devices, Inc., Norwood, Mass.). Current was injected by means of a high-speed, high-voltage amplifier (Burr-Brown Corp, 3584; JM). Total current was measured by means of a virtual ground circuit (Teledyne Philbrick, 1439; Teledyne Philbrick, Dedham, Mass.). The current measuring virtual ground electrode consisted of a large silver–silver chloride plate located across the bottom of the chamber. To eliminate polarization artifacts generated by injected current, an Ag-AgCl agar indifferent electrode was placed in the bath adjacent to the synapse. In most cases the time to plateau of the voltage microelectrode signal ranged from 50 to 150 μ s.

### ATP synthesis measurements using luciferin/luciferase

ATP synthesis was determined using luciferin/luciferase light emitting measurements (Mcelroy, 1947). Luciferase was pressure-injected into either the presynaptic or the postsynaptic terminal. Luciferin was added to the superfusate. Light emission was monitored and imaged using a single photon counting video camera (Argus −100, Hamamatsu Photonics). Light magnitude was determined using 15-s time integration periods.

### Block of ATP synthesis with oligomycin

Oligomycin (0.25 mg/ml) was injected presynaptically using 50–100 ms pressure pulses and visualized directly using the photon counting camera. The volume injected was in the range of 0.5–1 pl, i.e., about 5–10% of the presynaptic terminal volume (Llinas et al., 1991) for a final concentration of 25.0 μ g/ml, to block ATP synthesis.

### Ultrastructural methods

At the end of the electrophysiological recordings the stellate ganglion was immediately removed from the recording chamber and fixed by immersion in glutaraldehyde. Only synapses showing perfect preservation were accepted for analysis. Ultrastructural analysis was thus carried out on 240 active zones (AZ) from 8 synaptic terminals, as summarized in Table 1.

**Table 1:** **Number of synapses and active zones used in the ultrastructural analysis**.

**Electrophysiological study conditions and superfusate**	**Number of stellate ganglia (synapses)**	**Number of active zones analyzed/synapse**	**Total active zones analyzed**
Control ASW	2	40	80
Control ASW→RNS60	3	25–30	80
Presynaptic injection of oligomycin→RNS60	3	25–30	80
Totals	8		240

The tissue was postfixed in osmium tetroxide, stained in block with uranium acetate, dehydrated and embedded in resin (Embed 812, EM Sciences). Ultrathin sections were collected on Pioloform (Ted Pella, Redding, CA) and carbon-coated single sloth grids, and contrasted with uranyl acetate and lead citrate. Morphometry and quantitative analysis of the synaptic vesicles were performed with the Image J software (NIH, EUA). Electron micrographs were taken at an initial magnification of 20 or 30 K. They were enlarged on a computer screen to a magnification of 50 K for counting synaptic vesicles and to 75 K for counting clathrin-coated vesicles (CCV). Synaptic vesicle density and the number of CCV at the synaptic active zones were determined as the number of vesicles per μm^2^.

### Statistics

#### Morphology

The synaptic vesicle density was analyzed by the Kruskal-Wallis test (non-parametric test). Analyses were realized in the Statistical Analysis System Software 10.0 (Statistical Analysis System Institute Inc., EUA). The data is presented as average ± standard error).

#### Electrophysiology

Analysis of the electrophysiological data was carried out in the SPSS environment (SPSS Statistics, IBM). Several measurements of each parameter were made for each experiment. Statistical analysis was carried out on the grand mean of the mean for each synapse. The *t*-test or independent samples ANOVA followed by the Tukey *post-hoc* test were used to determine significance. Three statistical thresholds are marked, *P* < 0.05, *P* < 0.01, *P* < 0.001.

### Database

The data for this study were obtained from a total of 75 squid synapses yielding 81 experiments as summarized in Table 2. Synapses were included for analysis only if they had stable presynaptic and postsynaptic resting potentials and if the presynaptic and postsynaptic action potentials did not show signs of deterioration under control conditions.

**Table 2:** **Summary of experiments comprising database for this study**.

**Type of experiment**	**Control**	**Control RNS60**	**Control PNS50 RNS60**	^*^Oligomycin control RNS60	**Total**
Low oxygen content	–	10	–	–	10
Evoked release: single stimulus	–	5	–	–	5
Evoked release: recuperation from repetitive stimulation	4	9	5	7	25
Spontaneous release (noise analysis)	5	6	5	9	25
Presynaptic voltage clamp	Llinas et al., 1981b	6	–	–	6
Intracellular ATP generation (luciferin/luciferase)	–	10	–	–	10
Total	9	46	10	16	81

* Injected into presynaptic terminal.

## Results

### Electrophysiological studies

The initial experiments tested the ability of presynaptic activation to generate a postsynaptic response (Hagiwara and Tasaki, 1958; Takeuchi and Takeuchi, 1962; Kusano, 1968) in the presence of RNS60 ASW. In all of the synapses studied, superfusion with RNS60 ASW enhanced synaptic transmission. RNS60 ASW did not modify the resting membrane potential of the presynaptic membrane (Table 3). This was the case after intracellular injection of luciferase into the presynaptic terminal. RNS60 ASW did hyperpolarize the postsynaptic resting potential. This was most likely due to increased activity of the Na-K ATPase due to increased APT availability in the presence of RNS60. Membrane hyperpolarization was not seen when luciferase was injected into the postsynaptic terminal (Table 3).

**Table 3:** **Range and mean presynaptic and postsynatpic membrane resting potential in control ASW and RNS60ASW**.

	**Mean resting potential (mV) Range (mV) Number**	
**Superfusate**	**Presynaptic**	**Postsynaptic**
Control ASW	−60.0±0.23(−56.6, 61.8)(*n* = 19)	−59.9±0.16^*^(−54.8.0, −64.1)(*n* = 65)
RNS60 ASW	−60.6±0.89(−60.2, −60.9)(*n* = 9)	−60.7±0.21(−59.0, 64.3)(*n* = 43)
Lucifer control ASW	−59.7±0.50(−53.2, 62.0)(*n* = 17)	−59.5±0.61(−52, −61.8)(*n* = 17)
Luicfer RNS60 ASW	−60.0±0.33(−56.6, −62.0)(*n* = 17)	−60.3±0.40(−56.5, −62.3)(*n* = 17)

Postsynaptic control ASW vs. RNS60 ASW, independent samples t-test for equality of means.

* P < 0.01.

### RNS60 ASW rescues synaptic transmission from low oxygen block

**Figure 1:**
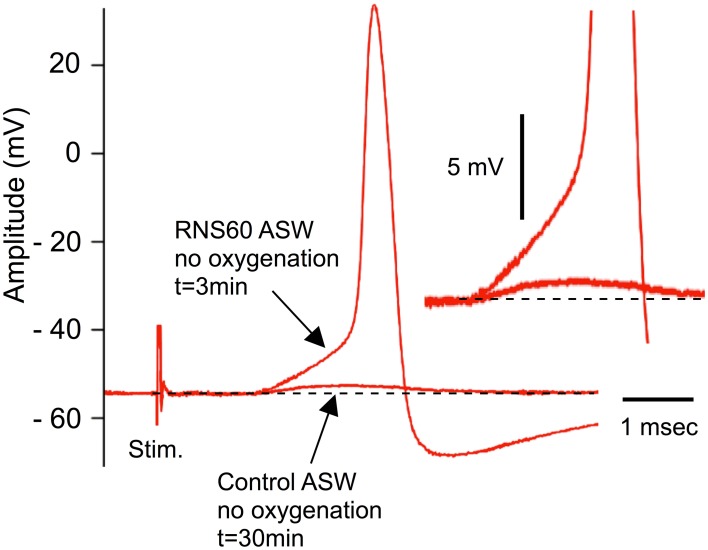
**Example of recovery of evoked transmitter release by RNS60 ASW in a hypoxic synapse following electrical stimulation of the presynaptic terminal.** Note small subthreshold synaptic potential after 30 min of hypoxia (lower arrow) and EPSP (upper arrow) and action potential elicited 3 min after superfusion with RNS60 ASW. Insert, amplitude magnification showing detail of the EPSP onset indicating change in amplitude without a change in release latency.

As originally demonstrated by Bryant (1958) and by Colton et al. (1992), synaptic transmission fails within 30 min when synapses are not properly oxygenated. This is due to transmitter depletion following hypoxia (Colton et al., 1992). Our initial set of experiments was designed to determine whether RNS60 could restore normal transmission in hypoxic synapses without having deleterious effects. These experiments consisted of allowing postsynaptic amplitude to decline such that only small, subthreshold postsynaptic synaptic potentials could be elicited (Figure 1, lower arrow). At that point, superperfusion with RNS60 ASW produced an increase in the postsynaptic potential, to the point that a postsynaptic spike could be easily evoked by each presynaptic stimulus. The action potential in Figure 1 was recorded 3 min after changing to RNS60 ASW. Such recordings could be made with long-term superfusion of RNS60 ASW, up to several hours. This demonstrates that RSN60 can rapidly and effectively restore transmission after hypoxic failure and does not itself have a deleterious effect on the transmission event as seen with oxygenated ASW (Colton et al., 1992).

### RNS60 ASW rescues transmission from high frequency stimulation synaptic fatigue

**Figure 2:**
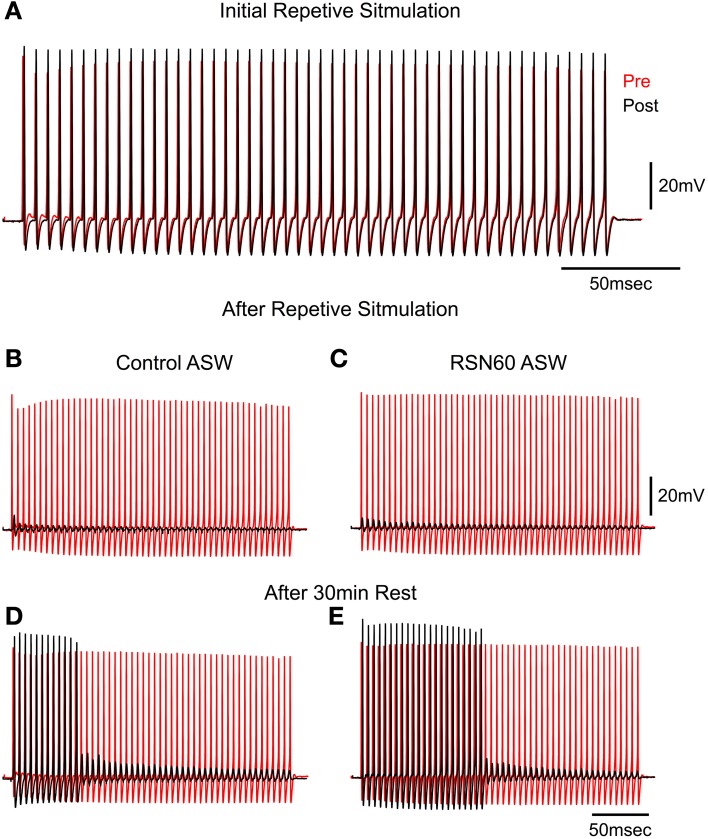
**High frequency stimulation in Control and RNS60 ASW. (A)** Presynaptic (red) and Postsynaptic (black) spikes generated by a repetitive presynaptic electrical stimulation at 200 Hz. **(B)** Failure of all postsynaptic spike generation after 100 consecutive trains repeated at 1 Hz in Control ASW. **(C)** Same as in **(B)** recorded in RNS60 ASW. **(D)** Partial recovery of postsynaptic spike generation after a 30 s rest period in control ASW. **(E)** Partial recovery after rest period in RNS60 ASW. Note in **(D,E)** that in the presence of RNS60 ASW there was a more vigorous recovery of postsynaptic spike generation after a similar 30 s rest period than in control ASW. Similar results were obtained in four other synapses utilizing the same stimulus paradigm.

Following the demonstration that no long-term deleterious changes occur with superperfusion with RNS60 ASW, a study of transmitter depletion following repetitive stimulation was carried out. High frequency stimulation of the squid giant synapse leads to a reduction of synaptic vesicles and failure of postsynaptic spike generation that can be restored after a period of rest (Kusano and Landau, 1975; Weight and Erulkar, 1976; Gillespie, 1979). This set of experiments was designed to test whether RNS60 altered the time course of recovery from such synaptic fatigue. Trains of 50 tetanic stimuli (at 200 Hz) were applied every second until synaptic failure (no postsynaptic spike) occurred. The synapse was then allowed to rest and the stimulus train was again applied. The number of spikes elicited during each train was used as an indication of transmission recovery, providing a quantitative measure of intracellular transmitter replenishment. This protocol was followed in control ASW and in RNS60 ASW, as shown in the example in Figure 2.

In control ASW the squid giant synapse can follow transmission at a stimulation rate of 200 Hz. As shown in Figure 2A, a 200 Hz stimulation train elicited a presynaptic action potential (red) and a postsynaptic action potential (black) for the first 49 of 50 stimuli. However, when stimulus trains were delivered at 1 Hz, transmission failed (no postsynaptic action potential) in control ASW (Figure 2B) and in RNS60 ASW (Figure 2C). A difference was seen, however, in the time course of recovery between control ASW and RNS60 ASW. In the example in Figures 2D,E, in control ASW after a 30 s rest period, the first 12 stimuli elicited postsynaptic spikes after which only subthreshold EPSPs were elicited (Figure 2D, 24% recovery). However, following superfusion with RNS60 ASW, the first 22 stimuli elicited a postsynaptic spike (Figure 2E, 44% recovery).

As this simple test allowed a first approximation methodology to test recovery from hypoxia, two types of experiments were implemented: (1) Recovery from repetitive stimulation in non-artificially oxygenated (control) ASW, or (2) recovery in the presence of RNS60 ASW. The mean recovery in control ASW was 14 ± 2.5% (*n* = 4) and that in RNS60 ASW was 68 ± 6.2% (*n* = 9). Statistical analysis revealed that the type of ASW had a significant effect on recovery [*T*_(1, 12)_ = 6.26, *p* < 0.0001].

These findings indicate that there was also an increase in transmitter availability in addition to an increase in the amount of transmitter (as indicated by the increased EPSP amplitude), during RNS60 ASW superfusion. This suggests that vesicular recycling may be modified, allowing rapid vesicular turnover and increased transmitter availability.

### RNS60 ASW increases spontaneous transmitter release

A related set of measurements of transmitter availability and release kinetics may be obtained by determining the magnitude of spontaneous transmitter release (Miledi, 1966; Kusano and Landau, 1975; Mann and Joyner, 1978; Lin et al., 1990) in the squid synapse. This measurement has often been utilized as a measure of vesicular availability at a given junction (Lin et al., 1990).

**Figure 3:**
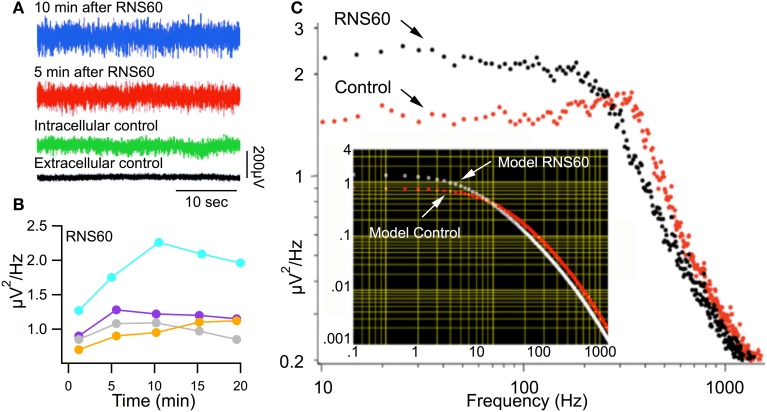
**Synaptic noise recorded in Control ASW and RNS60 ASW. (A)** Recordings showing synaptic noise across the postsynaptic membrane superfused with control ASW (green) and the increase in noise amplitude 5 min (red) and 10 min (blue) after superfusion with RNS60 ASW as well as the background extracellular noise recorded directly from the bath (black). **(B)** Plot of change in noise amplitude as a function of time after superfusion with RNS60 ASW in four synapses. **(C)** Plot of noise amplitude as a function of frequency (note log scale) in control ASW (red) and 10 min after superfusion with RNS60 ASW (black). The insert shows modeled results indicating that the change in noise could be interpreted as a change in the time course and amplitude of synaptic noise (for details see Lin et al., 1990).

In order to determine whether RNS60 can modify such spontaneous release, synaptic noise was measured in control ASW and after superfusion with RNS60 ASW (Figure 3). Figure 3A shows that synaptic noise recorded 5 min (Figure 3A, red trace) and 10 min (Figure 3A, blue trace) after superfusion with RNS60 ASW was greater than that recorded in control ASW (Figure 3A, green trace). Figure 3B is a plot of noise as a function of time after superfusion with RNS60 ASW for four synapses. Fast Fourier Transform (FFT) analysis of the synaptic noise showed that the increased spontaneous release occurred at frequencies over 200 Hz (Figure 3C). This is consistent with the function predicted by a model (Figure 3C, insert).

These results indicate a significant increase of spontaneous transmitter release, ranging from 20–80%. This increase reached a maximum about 10 min after changing from control ASW to RNS60 ASW. Such a result is shown for four synapses in Figure 3B where synaptic noise is plotted as a function of time after changing to RNS60 ASW. This increased level of spontaneous transmitter release was maintained for the duration of the experiments, up to 25 min, very much in accordance with the findings in Figures 1, 2.

### RNS60 increases transmitter release without increased presynaptic calcium current

The results discussed above indicate that superfusion with RNS60 ASW results in an increase in both evoked and spontaneous transmitter release that is possibly related to transmitter availability.

While such findings may be the result of any of the many components of the release process, one possible candidate is changes in presynaptic ionic channel kinetics following RNS60 ASW superfusion. Of these, the most likely would be modulation of presynaptic voltage-gated calcium current (ICa^++^). An increase in this parameter would explain many of the results described so far. Indeed, an increase in ICa^++^ would influence the degree of transmitter release by increasing the probability of vesicular fusion at the presynaptic terminals as well as an increase spontaneous transmitter release.

Given the possibility of implementing a presynaptic voltage clamp paradigm (Llinas et al., 1976, 1981a; Augustine and Charlton, 1986), this synapse is optimal as a research tool to address changes in presynaptic calcium currents.

A set of voltage clamp experiments was implemented to determine whether the RNS60 modulation of transmitter release seen above is mediated by an increase in the presynaptic calcium current. A second issue to consider was whether the relation between ICa^++^ and transmitter release (Llinas et al., 1981b) was maintained or otherwise modified by the presence on RNS60.

**Figure 4:**
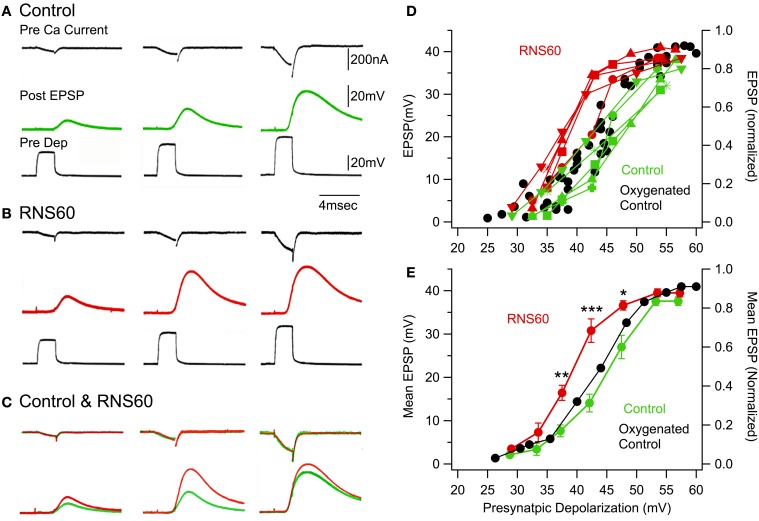
**Voltage clamp study indicating that RNS60 increases transmitter release without modifying calcium current or its relationship with transmitter release. (A)** Set of traces recorded in Control ASW show the amplitude and time course of the presynaptic calcium current (black), the amplitude and time course of the postsynaptic response (green) elicited by the rapid voltage clamp step shown in the third trace (Pre Dep, black). **(B)** Set of traces recorded in RNS60 ASW with the same amplitude depolarizing pulses as in the control set; EPSPs are red. **(C)** Superposition of calcium currents (upper traces) and EPSPs (lower trace) from panel **(A)** for control (green) and panel **(B)** for RNS60 (red) ASW demonstrates that there was no change in the time course or amplitude of the presynaptic calcium current, but a clear increase in the EPSP amplitude in RNS60 compared to control ASW. **(D)** Plot of EPSP amplitude as a function of presynaptic depolarization step for the five synapses. (Set of recordings from each synapse use the same marker.) The oxygenated control is modified from Figure 3B in (Llinas et al., 1981b) and provides data from seven synapses superfused with control ASW oxygenated with a 99.5% 02 and 0.5% CO_2_ gas mixture or with 0.001% H_2_O_2_. **(E)** Mean EPSP and s.e.m. as a function of mean presynaptic depolarizations for synapses in panel **(D)**. Oxygenated control is mean of data in Figure 3B in (Llinas et al., 1981b). [^*^T_(4, 8)_ = 4.27, *p* < 0.01; ^**^T_(4, 8)_ = 5.1, *p* < 0.001; ^***^T_(4, 8)_ = 3.54, *p* < 0.05, *t*-test].

Presynaptic calcium currents were elicited by graded depolarizing step pulses after pharmacological block of the voltage-gated sodium and potassium conductances (Llinas et al., 1976, 1981a; Augustine and Charlton, 1986). Figure 4A illustrates the presynaptic calcium current (Pre ICa), postsynaptic EPSP, and presynaptic voltage pulse (Pre Dep) at three levels of presynaptic depolarization in control (top traces, green) and RNS60 (bottom traces, red) ASW. The calcium current and EPSP traces are superimposed in Figure 4B. It is immediately apparent that the posstsynaptic response amplitude was larger in RNS60 (red) than in Control (green) ASW and that presynaptic inward calcium current was not significantly modified by RNS60. Note that the difference between the control and RNS60 EPSPs for the largest presynaptic depolarization is less than that for the middle depolarization. This is because the presynaptic membrane is close to the equilibrium potential for calcium, reducing ICa^++^ and the EPSP amplitude (Llinas et al., 1981a). The EPSP amplitude is plotted in Figure 4C for five synapses as a function of presynaptic voltage clamp depolarization. Each synapse has a different marker and the EPSPs recorded in Control ASW (green) RNS60 ASW (red) may be compared for each synapse. Note that the increase in transmitter release varied among synapses, but in every case was larger in the RNS60 ASW and reached a maximum value. Once this value was attained, we did not observe any further increase with protracted superfusion, suggesting that conditions for optimal transmitter release had been reached. When the mean amplitude of the postsynaptic response in control and RNS60 ASW were compared, significant differences were seen at three levels of depolarization. As may be seen in Figure 4D, depolarizing pulses were not exactly the same amplitude across synapses. To calculate the mean EPSP amplitude, the responses were assigned to one of four groups according to the presynaptic depolarization (two depolarization values, 16.5 and 25 mV, were not included a group). There was a significant difference in EPSP recorded in control and RSN60 ASW in three presynaptic depolarization groups: 38 mV, [*T*_(1, 8)_ = 4.27, *p* < 0.01]; 43 mV, [*T*_(1, 8)_ = 5.1, *p* < 0.001], 48 mV, [*T*_(1, 8)_ = 3.54, *p* < 0.01]. RNS60 did not change the decay constant of the EPSPs. This suggests that there was not a significant change in the passive properties (resistance or capacitance) of the postsynaptic membrane (τ, control 2.99 ± 0.7 ms; RNS60 2.36 ± 0.3 ms, *n* = 9).

Thus, the results from five voltage clamp experiments clearly indicate that the increase in transmitter release was not accompanied by a modification of calcium current kinetics or magnitude. At this point the possibility was considered that the effect of RNS60 could be related to some aspect of vesicular availability and related intracellular vesicular dynamics.

Of significance here is also the fact that when compared with similar voltage clamp results in past experiments (Llinas et al., 1981a,b) (Figures 4D,E, black) performed with oxygenated sea water, those results superimposed on our present control. This indicates that the increase in transmitter release following RNS60 based ASW increases transmitter release beyond that expected from normally oxygenated sea water.

### RNS60 increases ATP synthesis

This set of experiments was designed to determine the time course and magnitude of any change in ATP levels when the superfusate was changed from Control to RNS60 ASW. ATP levels were measured using the luciferin/luciferase protocol in which there is a direct correlation between light emission and ATP levels (Spielmann et al., 1981). Light measurements were made in both the presynaptic and postsynaptic elements of the synapse.

**Figure 5:**
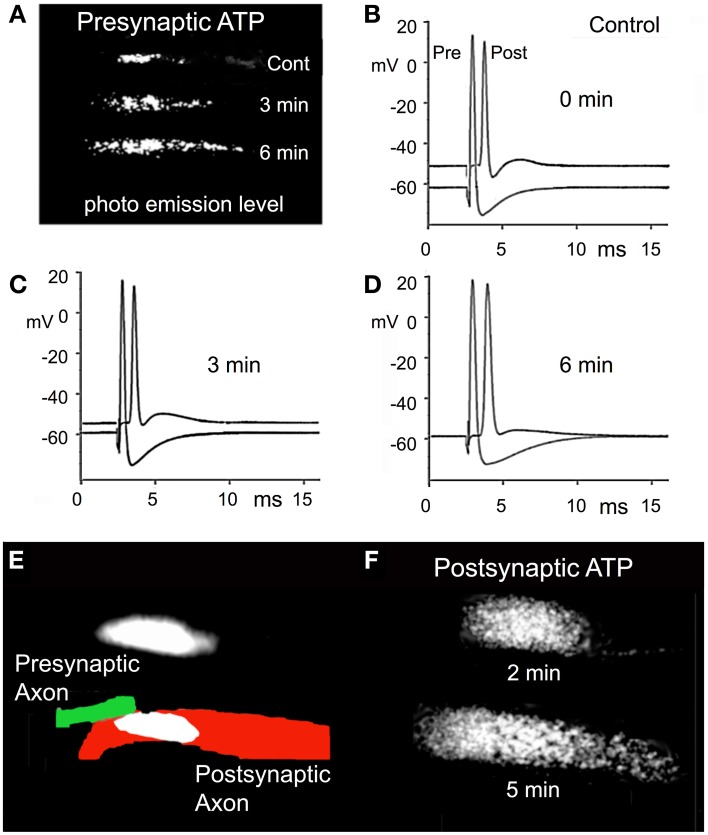
**Effect of RNS60 on ATP synthesis. (A)** The levels of luciferin/luciferase light emission in control ASW (Cont) and 3 and 6 min following RNS60 superfusion. **(B)** Presynaptic and postsynaptic action potentials in control ASW. **(C)** Action potentials recorded 3 min after superfusion with RNS60. **(D)** Action potentials recorded 6 min after superfusion with RNS60. **(E)** Drawing of presynaptic (green) and postsynaptic (red) element is superimposed on photograph of postsynaptic light emission. Presynaptic light emission is shown above the drawing. **(F)** Postsynaptic light emission 2 and 5 min after superfusion with RNS60. Note increase in postsynaptic resting potential in **(C,D)**, indicating an improvement of postsynaptic axon viability that is consistent with the increased level of ATP measured at the postsynaptic terminal following RNS60 ASW superfusion.

There was a clear increase in ATP levels from control levels, as indicated by the increased light emission recorded 3 and 6 min after the superfusate was changed from control to RNS60 ASW (Figure 5A). During this same period there was a small decrease in the resting potential of the presynaptic terminal, but no change in the action potential amplitude (Figures 5B–D). There was an increase in the resting potential in the postsynaptic axon from control levels at 3 min (Figures 5B,C). The resting potential continued to improve between 3 and 6 min after starting RNS60 superfusion. Unlike the presynaptic element, there was increase in the amplitude of the postsynaptic action potential (Figures 5B–D). An increase in postsynaptic ATP in the presence of RNS60 ASW is shown in Figures 5E,F. The results indicate that the increase in synaptic transmission following RNS60 superperfusion is accompanied by an increase in ATP levels in both the presynaptic and postsynaptic terminals.

### Block of ATP synthesis with oligomycin prevents effects of RNS60

One clear possibility to be addressed is whether the properties of the oxygen nanobubbles in RNS60 facilitated access of oxygen to intracellular compartments more efficiently than dissolved oxygen. If this were the case, one immediate possibility was that RNS60 ASW could support ATP synthesis more efficiently than diffusion-oxygenated ASW and thus increase vesicular availability either by increasing clathrin activity (Augustine et al., 2006) or by non-clathrin dependent vesicular endocytosis (Daly et al., 2000). Given this possibility, a set of experiments was designed to test whether blocking ATP synthesis by interfering with mitochondrial function (Jonas, 2004; Jonas et al., 2005) would prevent the modification of synaptic transmitter release by RNS60 ASW. A reduction of ATP would be expected to reduce transmitter release since many aspects of synaptic vesicle mobilization and recycling are mitochondrial ATP dependent (Talbot et al., 2003; Vos et al., 2010).

Mitochondria may be blocked with drugs that do not alter mitochondrial membrane potential (Ψ_*m*_), such as oligomycin or with depolarizing Ψ_*m*_ inhibitors. We did not use Ru360, an inhibitor of the mitochondrial uniporter because in some terminals Ru360 appears to inhibit Ca^2+^ influx across the plasma membrane (David, 1999). Mitochondrial depolarizing agents affect both ATP production and mitochondrial calcium uptake. It is proposed that most of the effects observed in synaptic transmission by depolarizing Ψ_*m*_ inhibitors are related to changes in calcium dynamics at the presynaptic terminal (Billups and Forsythe, 2002; Talbot et al., 2003). We chose to use oligomycin, which inhibits ATP synthase but does not depolarize mitochondria, and is reported to have no effect on either cytosolic or mitochondrial calcium dynamics in several preparations, but instead acts by blocking complex V (David, 1999).

The most sensitive measure of vesicular turnover and the overall release apparatus is spontaneous transmitter release, since it involves the least number of steps in its activation. With this in mind, we implemented a set of experiments in which we determined the effect of blocking ATP synthesis on spontaneous transmitter release.

**Figure 6:**
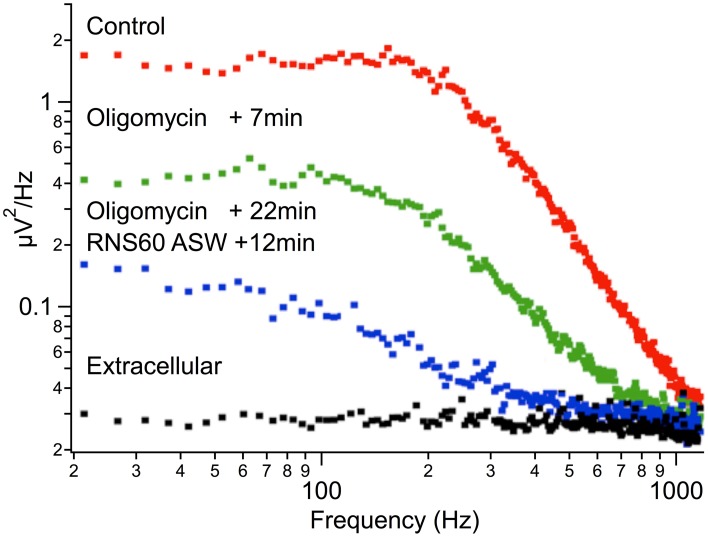
**Reduction of spontaneous synaptic release following oligomycin administration.** Plot of noise amplitude as a function of frequency (note double log coordinates). Red, control ASW; green, 7 min after addition of oligomycin; blue, 22 min after oligomycin administration and 12 min after changing superfusate to RNS60 ASW. Black, extracellular recording.

Presynaptic intracellular oligomycin injection (0.25 mg/ml) during control ASW superfusion markedly reduced spontaneous release from control levels (compare Figure 6, red vs. green). This occurred rapidly in all experiments. A reduction of more than an order of magnitude occurred within the first 7 min after oligomycin injection into the presynaptic terminal. Changing the superfusion to RNS60 ASW 22 min after injection of oligomycin failed to increase spontaneous transmitter release (Figure 6, blue trace, recorded 12 min after the start of RNS60 ASW superfusion). Similar findings were noted in 5 experiments. Thus, RNS60 ASW failed to rescue synaptic transmission from the reduction caused by ATP depletion.

### Ultrastructural analysis of RNS60 ASW superfused synapses

Electron microscopic analysis of presynaptic and postsynaptic morphology revealed very well preserved ultrastructural changes following RNS60 ASW administration. In general terms, the ultrastructure demonstrated well-preserved cytosolic properties as well as mitochondrial profiles (Figures 8, 9). The number of synaptic vesicles and CCV were analyzed in 1 μm^2^ of each active zone. Quantification was carried out in 25–30 active zones in 2 control synapses and 3 RNS60 ASW synapses.

### RNS60 ASW modifies vesicles at synaptic active zones

Three main differences from normal morphology were noticed at the synaptic active zone following RNS60 administration: (1) a reduction of the number of lucid, regular-sized synaptic vesicles (SSV), (2) an increase in the number of clathrin–coated vesicles (CCV), and (3) an increase in the number of irregular large diameter vesicles (LEV), suggesting increased release dynamics.

**Figure 7:**
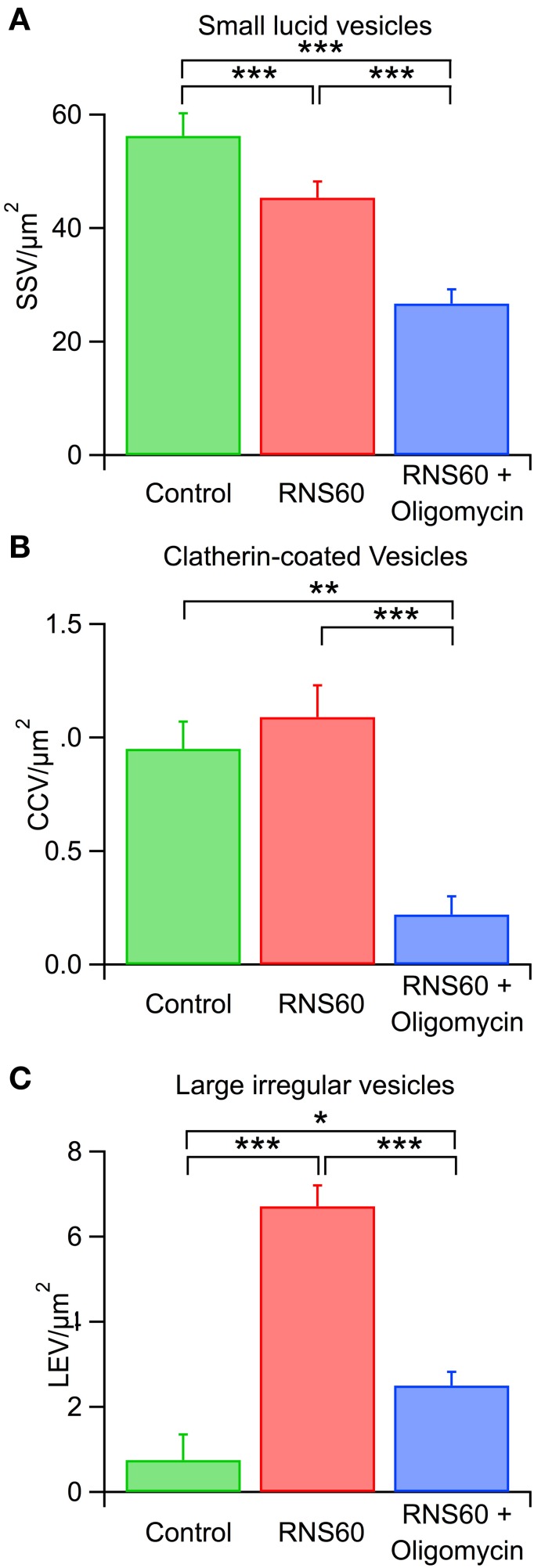
**Effect of RNS60 and oligomycin on synaptic vesicle numbers. (A)** Number of lucid small synaptic vesicles after superfusion with control (green), RNS60 (red), or RNS60 after presynaptic injection of oligomycin (blue) in the presence of RNS60. **(B)** Number of clatherin-coated vesicles under the same three conditions as in panel **(A)**. **(C)** Number of large, irregular vesicles under the same three conditions as in panel **(A)**. ^***^p < 0.0001, ^**^p < 0.001, ^*^p < 0.05, Kruskal-Wakkus test.

There was a statistically significant decrease in SSV number in RNS60 ASW superfused terminals compared with control terminals (Figure 7A, red and green). By contrast, the number of CCVs was higher in RNS60 than control (Figure 7B, red and green) synapses but this difference did not reach significance. In addition, a large increase in the number of large vesicles (Figure 7C, red and green) suggests an increased vesicular turnover, as would be expected from an increased ATP level at the presynaptic terminal. These results are in accordance with our research on the relation between mitochondria and vesicular formation and availability (Ivannikov et al., 2013).

**Figure 8:**
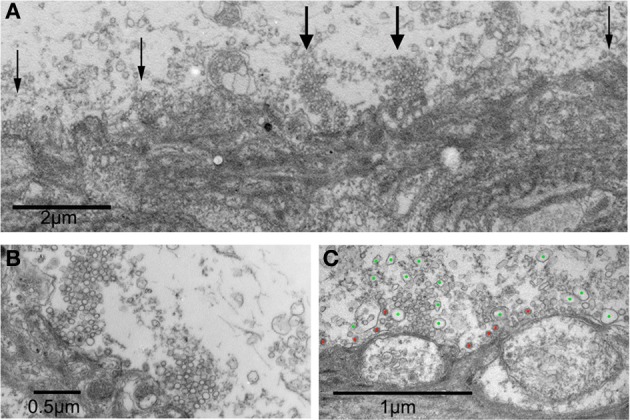
**Electronmicrographs of a synaptic junctions following RNS60 ASW superfusion. (A)** Presynaptic and postsynaptic image at low magnification showing postsynaptic digit making several contacts forming active zones with the presynaptic terminal (arrows) in a synapse superfused with control ASW. **(B)** Higher power image showing two active zones from panel **(A)** that are marked with wide arrows. **(C)** Vesicles of irregular shapes and sizes are present in the terminals from a synapse superfused with RNS60 ASW. Green dots denote large synaptic-like vesicles, red dots mark clathrin-coated vesicles.

These differences are illustrated in Figure 8. In Figure 8A a postsynaptic digit emerges from the postsynaptic axon to form several contacts with the presynaptic terminal in a synapse after control ASW. The active zones are marked with arrows. Figure 8B illustrates two active zones from Figure 8A at a higher magnification. Figure 8C is two active zones from a synapse superfused with RNS60 ASW. Note the high number of large synaptic vesicles (blue dots) and CCV (red dots) compared to the control AWS active zones.

### Block of ATP synthesis with oligomycin prevents effects of RNS60

**Figure 9:**
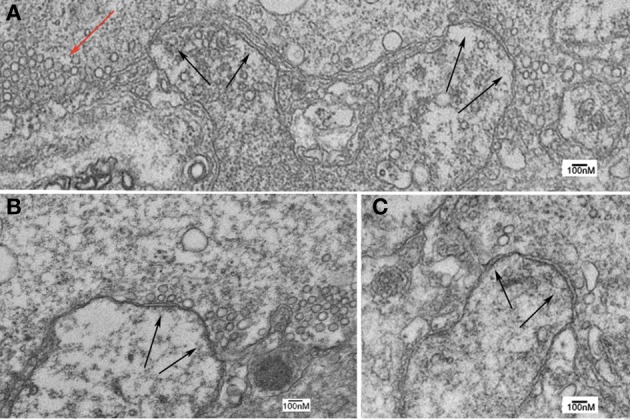
**Ultrastructure of squid giant synapse active zones following oligomycin injection. (A–C)** Black arrows indicate active zones showing few, if any, synaptic vesicles. Note also the lack of clathrin-coated vesicles and of large vesicular profiles that are generally found in the presence of synapses superfused with RNS60 ASW. Note also the presence of a few vesicles scattered away from the active zone (red arrow).

In synapses in which the presynaptic terminal was injected with oligomycin in the presence of RNS60, the results from ultrastructural analysis indicate a significant reduction in all synaptic vesicle types compared to controls and to RNS ASW without Oligomycin injection (Figure 7). Indeed, images from such synapses (Figure 9) indicate that while the ultrastructure is not grossly altered, the numbers of vesicles of all types in the vicinity of the active zones are very much reduced.

## Discussion

It is clear from the data presented that RNS60, a physically modified saline containing charge-stabilized nanostructures, has significant function-enhancing properties. The importance of these findings concerns the fact that RNS60 represents a novel class of bioactive agents relating to physical properties of solutions rather than added chemical molecules. RNS60 has shown cytoprotective and anti-inflammatory effects in different models of neurodegeneration through direct effects on glial cells as well as modulation of T cell subsets (Khasnavis et al., 2012; Mondal et al., 2012). Together with the results described here, this suggests that RNS60 exerts pleiotropic effects that are not based on interaction with a specific receptor, but rather that RNS60 is a facilitator of physiological function that requires a different appellative. RNS60 did not have the same level of effect in all synapses. Rather, although the results were always in the same direction, the degree varied among synapses, as seen in Figure 3B. This is related, we propose, to the initial state of the synapse and is a subject of future studies.

Functionally, the current studies demonstrate that RNS60 is able to enhance synaptic transmission without affecting normal function and without any deleterious side effects, as has been demonstrated in previous studies in other systems including human use (Khasnavis et al., 2012; Mondal et al., 2012). RNS60 ASW did hyperpolarize the postsynaptic resting potential (Table 3). This was most likely due to increased activity of the Na-K ATPase following increased APT availability in the presence of RNS60.

Indeed, the results concerning single spike synaptic transmission (Figure 1), as well as the response to repetitive presynaptic terminal activation (Figure 2), indicate that the ability of RNS60 to maintain and enhance synaptic transmission within normal parameters is not accompanied by abnormal responses. This indicates the absence of overdose or side effects within the time course of these experiments. Such a conclusion is also supported by the increase in spontaneous release that reaches a maximum level following a single superfusion of RNS60 for a period of 30 min and decays slowly after superfusion with Control ASW.

Concerning the mechanism of action of RNS60, the possibility that it could be modifying channel kinetics, and in particular calcium currents, was discarded by the voltage clamp results, which indicate that the increased postsynaptic response was not correlated with any change in the time course or amplitude of the inward calcium current responsible for the transmitter release (Figure 4).

An alternative explanation for the increased postsynaptic responses lies in changes in the postsynaptic membrane resistance or resting potential. To address a possible membrane resistance change, we compared the time course of the falling phase of the EPSP in control and RNS60 ASW since this depends on the RC properties of the membrane. As there were no differences in this parameter, that possibility is excluded. Similarly, although a difference was sometimes seen in the resting potential, as illustrated in Figure 5, this was an exception as the postsynaptic membrane potential and spike amplitude were both below average in this synapse, probably due to an initial damage of the postsynaptic axon during dissection. This was not generally seen and the resting potential differences observed in all other synaptic recordings did not reach significance (Table 3).

It may be concluded, therefore, that RNS60 does change available energy level via ATP increase, and that this is accompanied by an increase in synaptic transmission effectiveness (Figure 5). In addition, an unexpected finding was that of the noise frequency change in the presence of RNS60 (Figure 3C). The fact that, at the level of spontaneous release, there is a clear change in the noise profile seen as a reduction of high frequency noise and an increase of low frequency noise (Figure 3C), seems to correlate with the change in the synaptic vesicle size distribution (Table 3, Figure 8). One possibility that must be considered in future research is that the transmitter delivery kinetics may be different between normal vesicular profiles and that of the larger endosome related vesicles. The latter could have a slower release kinetics that may explain the change in noise frequency toward lower frequency with an accompanying noise level amplitude increase.

As shown in RNS60 superfused terminals, large vesicles with different shapes and sizes were observed (Figure 7C). These structures were rarely observed in control synapses (Figure 7A) or in terminals studied in former experiments (Heuser and Reese, 1973).

Neurotransmitter release requires a well-known set of steps concerning synaptic vesicle exo- and endocytosis (Heuser and Reese, 1973). It has been shown in previous work that dynamin/synaptophysin complex disruption results in a decrease of transmitter release resulting from a depletion of synaptic vesicle recycling (Daly et al., 2000). It was also observed that, under these conditions, the number of CCVs actually increased, suggesting the existence of another vesicle endocytosis mechanism with a faster time course than the classical clathrin pathway (Daly et al., 2000). This finding was further corroborated by the injection of Rabfilin 3A and/or one of its fragments which affect the distribution of membranes of the endocytotic pathway in the squid presynaptic terminal in a multifunctional fashion (Burns et al., 1998). This is consistent with previous observations following different domains manipulation of the synaptic vesicle protein synaptotagmin (Fukuda et al., 1995; Mikoshiba et al., 1995). Increased expression of the brain vesicular monoamine transporter VMAT2 regulates vesicle phenotype and quantal size (Pothos et al., 2000).

While enhanced synaptic transmission following RNS60 administration was not accompanied by a significant change in the number of active zones, a number of large, lucid vesicles (up to 300 nm in diameter) were observed in the immediate vicinity of the active zone. These could be part of the enhanced synaptic transmitter release that is observed under these conditions (Figures 7C, 8). Since such vesicles are both large and are not surrounded by a clathrin mesh, this suggests that the endocytotic mechanism responsible for their presence may be independent of the clathrin or caveolin pathway, as previously reported (Burns et al., 1998) and reviewed in detail (Mayor and Pagano, 2007).

The fact that both spontaneous release levels as well as the amplitude of the evoked synaptic potentials are increased significantly suggests that the release of the larger vesicular component may be increased. Such a change in the distribution of vesicular size, favoring the larger endosomal vesicular profiles over the smaller clathrin related vesicles, confirms a similar morphological analysis of vesicular size distribution following high level synaptic activation as reviewed by Saheki and De Camilli (2012).

This change in vesicular size distribution provides a possible explanation for the fact that the nature of the spontaneous synaptic noise was modified after RNS60 ASW administration, as shown in Figure 3 and as discussed in the description of synaptic noise and its relation to time course of synaptic miniature potentials and vesicular size.

Mitochondria are energy-supplier organelles, strikingly abundant in chemical synapses (Palay, 1956; Talbot et al., 2003). In squid, the presynaptic terminal mitochondria lie in close juxtaposition to presynaptic calcium channels (Pivovarova et al., 1999). Energy supply to neurons in the form of oxygen and glucose and its final product—mitochondrially generated ATP—is largely used for reversing the ion influxes underlying synaptic and action potentials (Attwell and Laughlin, 2001). Here we tested whether inhibition of mitochondrial ATP with oligomycin modified the effect of RNS60 on synaptic transmission.

Concomitant application of RNS60 and the complex V mitochondrial blocker oligomycin failed to induce increments in spontaneous release as determined by synaptic noise power spectrum analysis (Figure 6). These experiments suggest that RNS60's mechanism of action is dependent on mitochondrial ATP production, possibly by providing oxygen in a more efficient manner.

In conclusion, concerning the mechanism of action of RNS60, a significant result relates to the finding that a block of mitochondrial ATP synthesis results in inactivation of the RNS60 effect on synaptic transmission. These findings further indicate that RNS60 does not operate directly on the vesicular release mechanism but rather indirectly via an increased synthesis of ATP by the mitochondrial system (Figure 5). This has been shown to have a significant effect on both the availability of vesicular organelles and on their movement on to the active zone at the presynaptic compartment in the synaptic junction region (Ivannikov et al., 2013).

We propose, therefore, that RNS60 enhanced ATP synthesis by facilitating oxygen transport into the mitochondrial system and that it does so with minimal increase in intracellular free radical level. The fact that RNS60 can increase ATP synthesis is significant beyond synaptic transmission, in that it suggests the possible therapeutic application of this modified saline in disorders related to cellular energy metabolism, as recently proposed for Alzheimer's Disease (Cavallucci et al., 2013).

## Author contributions

Rodolfo R. Llinás designed the experiments. Rodolfo R. Llinás, Mutsuyuki Sugimori, Soonwook Choi, Eunah Yu, Guilherme Rabello, Herman Moreno, performed the electrophysiology experiments. Rodolfo R. Llinás, Mutsuyuki Sugimori, Soonwook Choi, Eunah Yu, and Kerry D. Walton analyzed the electrophysiological data. Jorge E. Moreira, Ajmal Zemmar, Suelen Merlo performed and analyzed the morphological experiments. Rodolfo R. Llinás and Kerry D. Walton prepared the manuscript. All authors contributed to editing and approved the manuscript.

### Conflict of interest statement

The authors declare that the research was conducted in the absence of any commercial or financial relationships that could be construed as a potential conflict of interest.
